# Supplementary material for: National Facilitators and Barriers to the Implementation of Incentives for Antibiotic Access and Innovation
Source: Antibiotics (Basel). 2021 Jun 21;10(6):749. doi: 10.3390/antibiotics10060749 (PMC8234425; doi:10.3390/antibiotics10060749)
Supplement: Supplementary file 1 [file antibiotics-10-00749-s001.zip › antibiotics-1254438-supplementary.pdf]

Feedback regarding incentives for antibiotic access and innovation

INTERVIEW TEMPLATE

General aims of the interviews and handling of data

- Chatham House Rule - When a meeting, or part thereof, is held under the Chatham House Rule, participants are free to use the information received, but neither the identity nor the affiliation of the speaker(s), nor that of any other participant, may be revealed.
- All data gathered through these country visits will be handled confidentially and anonymously. All answers will be understood to represent expert feedback and not government commitment.

- The purpose of these country visits is to gain a better understanding of the diversity of country perspectives on the opportunities and barriers to ensuring access to important old and new antibiotics (both for human and animal health), incentives for improved infection prevention and control, as well as incentives for stimulating antibiotic innovation. We anticipate that the insights gathered from these interviews will be used to inform the design of more granular incentives and mechanisms appropriate to, and more aligned with, country expectations.
- The focus of the interviews is regarding securing predictable access to important antibiotics (both old and new), incentives to improve infection prevention and control, and incentives to support research and innovation of new antibacterial treatments.
- The results will be synthesized and published in an open access peer-reviewed journal.
- We are not seeking government commitment, only your valuable feedback.

Interviews with AMR experts in human health

1. Introductions

2. Aims of the meeting and handling of the data gathered (see page 1)

3. Could you please briefly describe your national healthcare system, including how medicines are reimbursed?

4. Could we please discuss your country's areas of greatest concern or vulnerability regarding AMR research, innovation, and access?

5. Has your country assessed the cost and/or burden of AMR?

6. Incentives/measures to maintain access to existing (and typically older generic) antibiotics
   - Is your country experiencing shortages of important antibiotics (including older generic antibiotics)?
   - What steps is your country pursuing (if any) to maintain predictable access to these antibiotics?

- Which antibiotics are you most concerned about? Do you have a list of critical antibiotics?
- Does your country aim to utilize more narrow-spectrum antibiotics in order to reduce broad-spectrum consumption?
- Is there a willingness to pay higher unit prices for older antibiotics to secure availability? Or what other incentives might your country be interested in? Could your country be interested in testing new incentives within well-defined parameters and financing constraints? What are the barriers and influencing factors?
- Would your country prefer working multilaterally, for example by pooling funds with other countries? If so, which facilities are considered the strongest candidates for a pooled fund? About how many other countries would need to commit?
- What might be the first steps towards a pilot?

7. Incentives/measures to support Infection Prevention and Control (IPC)
   - OECD has demonstrated that significant cost savings can be achieved by improving IPC measures. How relevant do you feel that this is in your country?
   - If so, these often require upfront financing to achieve the savings. Are there mechanisms in your country to finance these efforts?
   - Could economic incentives be useful for infection prevention and control? For example, upfront financing in line with expected outcomes, followed by sharing of cost savings between the healthcare institution and government?

8. Incentives for new antibiotics and other treatments

   - What steps is your country pursuing (if any) to secure access to new antibiotics (or other treatments)? Are you concerned that your country will not have access to the newest antibiotics?
   - How would you gauge the level of awareness among your government policymakers regarding the potential unavailability of effective antibiotics due to shifts in bacterial resistance?
   - Are your government policymakers aware of the existing literature regarding stimulating antibiotic innovation (for example, the UK's AMR Review, DRIVE-AB, etc.)? How relevant do they perceive these measures are for your country?
   - Does your country feel that the priority pathogens identified by WHO are aligned with your country's unmet public health need?
   - Might there be interest from your country to test incentives aligned with different needs or urgencies, for example based on WHO's AWARE classification?
   - Might your country be interested in working multilaterally, for example by pooling funds with other countries? If so, which facilities are considered the strongest candidates for a pooled fund? About how many other countries would need to commit?
   - What might be the first steps towards a test?

9. What financing mechanisms could be used to finance the abovementioned incentives?

10. Meeting wrap-up and next steps
